# Vertebrate-wide transcriptomic screening identifies immune cell-specific expression of the conserved OR-κ gene

**DOI:** 10.1186/s40851-026-00260-z

**Published:** 2026-02-07

**Authors:** Kanoko Nishiura, Tatsuki Nagasawa, Masato Nikaido

**Affiliations:** School of Life Science and Technology, Institute of Science Tokyo, Tokyo, Japan

**Keywords:** Chemoreceptor, Olfactory receptor, Ectopic expression, Evolutionary biology

## Abstract

Vertebrate chemoreceptor genes play a central role in detecting environmental chemical compounds through the olfactory organs and taste buds, enabling the perception of odors and tastants that are critical for survival. Through evolutionary processes, these genes have repeatedly undergone duplication, divergence, and pseudogenization, giving rise to lineage-specific gene repertoires that reflect ecological and behavioral adaptation. While the canonical functions of these chemoreceptors are confined to the nasal and oral cavities, accumulating evidence, particularly in mammals, indicates that some chemoreceptors are expressed and function in non-chemosensory (extra-nasal/oral) organs. However, such extra-nasal/oral expression has rarely been examined from a broad evolutionary perspective across vertebrate lineages. Here, we systematically investigated organ-wide expression patterns of chemoreceptor genes by conducting comprehensive bulk RNA-seq analysis across 13 organs in four representative species: mouse, *Xenopus*, *Polypterus*, and zebrafish. In all species, the majority (95–97%) of chemoreceptor genes were expressed in olfactory and gustatory organs, as expected. Remarkably, a subset (1–29%) showed expression in extra-nasal/oral organs, suggesting that such extra-nasal/oral expression may be a common phenomenon across vertebrates. In particular, the evolutionarily conserved OR-κ gene, with stable gene copy numbers, exhibited organ-independent expression across all analyzed species. Single-cell RNA-seq data further revealed that OR-κ is predominantly expressed in immune cells, implying potential function of chemoreception in immune systems. Furthermore, genomic context analysis showed that the OR-κ gene is isolated from canonical OR gene clusters, suggesting it may have distinct transcriptional regulatory mechanisms compared to typical olfactory receptors. Our findings expand the conventional view of chemoreceptors as sensory-specialized molecules, highlighting their unexpected functional diversity across vertebrate organs. Notably, the OR-κ gene appears to have an ancient evolutionary origin that likely traces back to the common ancestor of vertebrates. Taken together, this study provides us compelling evidence to reconsider the functions and evolutionary trajectories of chemoreceptor genes in vertebrates.

**Supplementary Information:**

The online version contains supplementary material available at 10.1186/s40851-026-00260-z.

## Background

The accurate perception of the surrounding environment is essential for animal survival, allowing individuals to recognize nutritional sources, toxins, predators, and reproductive partners [1]. Among vertebrate sensory systems, olfaction and gustation are collectively referred to as chemosensation, because they detect chemical compounds derived from the external environment [2]. In vertebrates, chemoreception in the nasal cavity (olfactory organ/epithelium or vomeronasal organ) and oral cavity (including tongue) is mainly mediated by a subclass of chemoreceptors belonging to the G protein-coupled receptor (GPCR) superfamily [3, 4]. These GPCRs comprise six different multigene families encoding seven-transmembrane-domain receptors: olfactory receptors (ORs), trace amine-associated receptors (TAARs), vomeronasal receptor types 1 and 2 (V1Rs and V2Rs), and taste receptor types 1 and 2 (T1Rs and T2Rs) [5].

Chemoreceptor genes were initially discovered in mammalian nasal/oral organs as candidate molecules for odorant and gustatory perception [6–13]. Subsequently, homologous genes have been successively identified in non-mammalian vertebrates [14–18], and their expression in nasal/oral organs has been demonstrated in many species, such as channel catfish [14], zebrafish [16, 17, 19–21] and African clawed frog [22, 23]. Recent advances in whole-genome sequencing technologies and the resulting expansion of genomic data have illuminated the evolutionary history of vertebrate chemoreceptor genes. OR, TAAR/V1R/V2R, and T1R/T2R genes were acquired before the divergence of cnidarians and bilaterians [24], jawless and jawed fish [25–30], and jawed vertebrates [31–33], respectively. These findings indicate that chemoreceptor genes, while first discovered in mammals, are evolutionarily ancient and have been conserved across diverse vertebrate lineages [27, 34–36].

Vertebrate chemoreceptor genes are encoded as multigene families distributed across multiple genomic loci. During vertebrate evolution, these gene families have undergone repeated gene duplication and pseudogenization events—a process known as birth-and-death evolution—resulting in lineage- and species-specific variation in gene copy numbers. For example, the highest known copy numbers are reported for ORs (2,399 in echidna, *Tachyglossus aculeatus*), TAARs (497 in reedfish, *Erpetoichthys calabaricus*), and T2Rs (268 in frog, *Glandirana rugosaa*) [37–41]. Such continuous turnover of chemoreceptor genes through the birth-and-death process generates extensive diversity in receptor-specific amino acid sequences, contributing to receptivity across a wide range of chemical compounds [42]. Thus, the diversity of chemoreceptor genes across lineages is thought to facilitate the precise discrimination and perception of the myriad environmental chemical compounds encountered in each species’ habitat [37].

Some of these multigene families encoding chemoreceptor genes are known to function in organs beyond the nasal and oral organs. For instance, human *OR17-4* and mouse *OR23*, both expressed in sperm, mediate chemotaxis in response to small chemical molecules or short peptides [43, 44]. Additionally, human and mouse *T1R2* and *T1R3* have been associated with maintaining intestinal glucose homeostasis by regulating the sodium-glucose co-transporter SGLT1 [45, 46]. Moreover, the secretion of gastrointestinal hormones can be modulated through the activation of mouse *T2R108* receptor by luminal ligands [47, 48]. Thus, chemoreceptor genes expressed in extra-nasal/oral organs appear to be used for monitoring the internal rather than the external environment [49]. In several studies, these receptors are referred to as “ectopic olfactory receptors” or “ectopic taste receptors”, terms generally implying abnormal expression in non-chemosensory organs [50, 51]. However, chemoreceptors expressed in non-chemosensory organs are being increasingly discovered [45, 52–59], and are now recognized as a widespread phenomenon. Therefore, in this study, we refer to these as “extra-nasal/oral chemoreceptors” rather than ectopic chemoreceptors. While expression patterns of chemoreceptor genes in extra-nasal/oral organs have been increasingly characterized in mammals, particularly humans and mice, knowledge from non-mammalian species remains limited, with only a few examples reported in zebrafish [60, 61] and blind cavefish [62]. Consequently, a comprehensive evolutionary perspective has been lacking.

Recently, bulk and single-cell RNA-seq data from various organs have become available, providing new opportunities to investigate the molecular evolution of chemoreceptor genes across a broad range of vertebrates. In this study, we conducted a comprehensive screening of chemoreceptors exhibiting extra-nasal/oral expression in representative vertebrates: mouse, *Xenopus*, *Polypterus*, and zebrafish. As a result, we detected the expression of members of all six chemoreceptor gene families in extra-nasal/oral organs, suggesting that such expression may represent a common feature shared across vertebrates, from fishes to mammals. Furthermore, we found that OR-κ, which is evolutionarily stable compared to the expanded OR families, is predominantly expressed in immune cells in both teleost fishes and mammals. Given that OR-κ originated before the divergence of jawless fishes, these findings highlight the need to reconsider the function and evolutionary origins of vertebrate chemoreceptor genes.

## Results

### The exploration of extra-nasal/oral expression of chemosensory genes from bulk RNA-seq

**Fig. 1:**
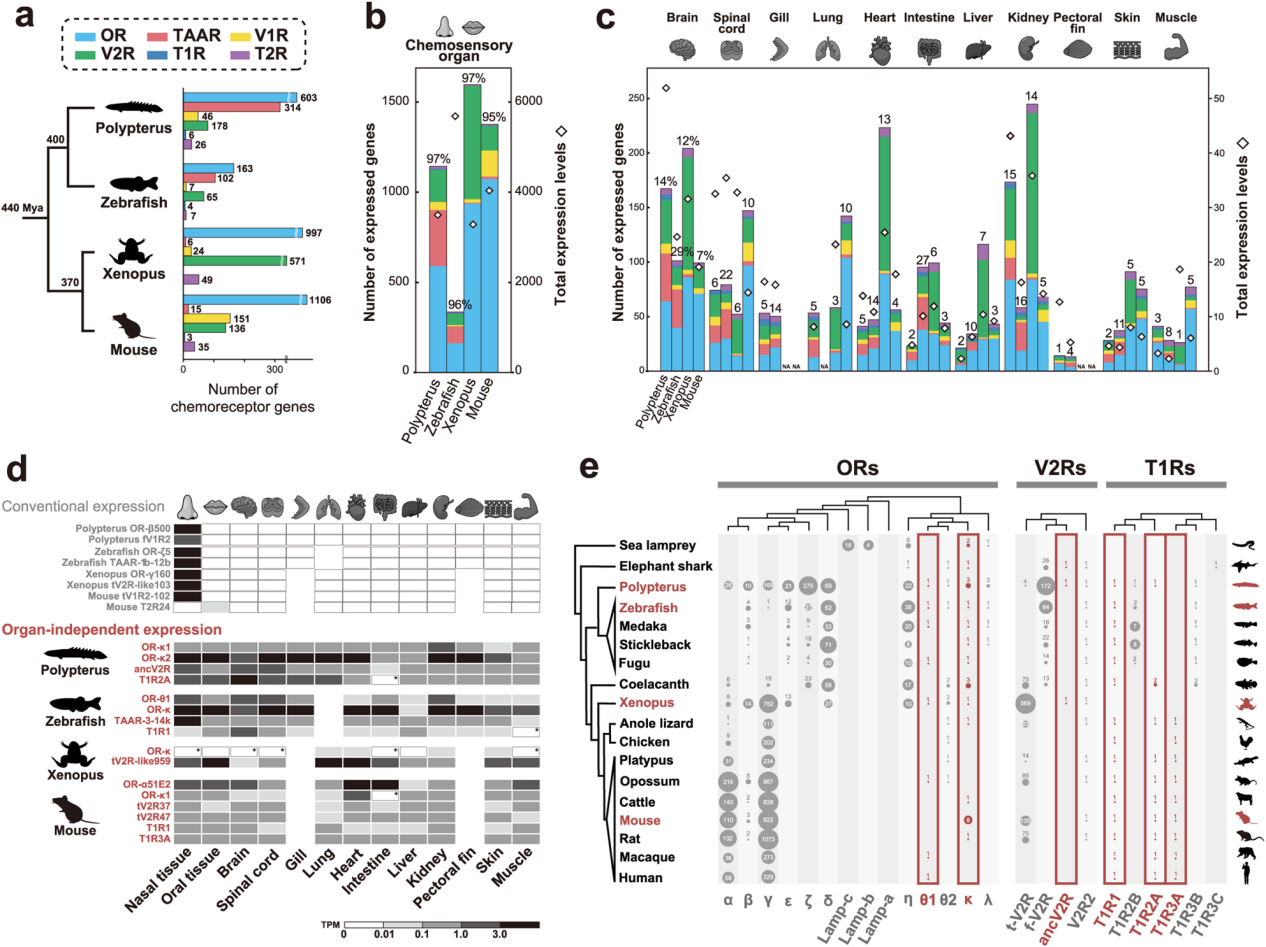
Transcriptomic detection of chemoreceptor genes from bulk RNA-seq data.(a) Number of chemoreceptor genes used in this study. All sequence information for chemoreceptor genes was obtained from previous studies [38]. Numbers at the nodes in the species tree indicate divergence dates (Mya, million years ago). (b) Number, proportions (relative to total chemoreceptor genes), and total expression levels of genes detected in chemosensory organs (nasal/oral organs). Bar colors correspond to those in Fig. 1a. (c) Numbers, proportions, and total expression levels of genes detected in extra-nasal/-oral organs. Bar colors correspond to those in Fig. 1a. (d) List of genes expressed in multiple organs. While conventional chemoreceptor genes showed chemosensory organ-specific expression, several genes were detected with organ-independent expression. The asterisk indicates that expression was detected by RT-PCR rather than bulk RNA-seq. (e) Organ-independently expressed genes were not members of expanded chemoreceptor subfamilies. Subfamily classification of each chemoreceptor gene (above phylogenetic tree) followed previous studies [21]. The number of genes contained in each subfamily is indicated by a bubble chart labeled numerically. Genes that exhibited organ-independent expression were not conventional expanded chemoreceptor genes but rather evolutionarily conserved across species (red boxes). Species analyzed by bulk RNA-seq in this study are highlighted in red. Some animal silhouettes were obtained from PhyloPic.org (full links for each silhouette can be found in Table S11).

First, we conducted bulk RNA-seq analysis of 13 organs to obtain a comprehensive overview of chemoreceptor gene expression across vertebrate species (Fig. 1 and Table S2–5). We analyzed a mammal (mouse: *Mus musculus*), amphibian (Western clawed frog: *Xenopus tropicalis*), basal ray-finned fish (bichir: *Polypterus senegalus*), and teleost fish (zebrafish: *Danio rerio*). The gene annotation dataset of chemoreceptors was compiled based on previous studies [37] (Fig. 1a). All bulk RNA-seq results in this study are summarized in Fig. S1. The majority of chemoreceptor genes (97% in *Polypterus*, 96% in zebrafish, 97% in *Xenopus*, and 95% in mouse) were expressed in nasal/oral organ, which serve as canonical chemosensory tissues containing olfactory neurons and taste bud cells (Fig. 1b). In contrast, a subset of genes (ranging from 1% in the *Polypterus* pectoral fin and the *Xenopus* muscle to 29% in the zebrafish brain) also exhibited expression in extra-nasal/oral organs. Although most chemoreceptor genes were primarily expressed in chemosensory organs, all analyzed species possessed a subset of genes expressed in extra-nasal/oral organs, which may reflect that extra-nasal/oral expression is a widespread and evolutionarily conserved feature among vertebrates (Fig. 1c).

Among the chemoreceptor genes showing extra-nasal/oral expression, some genes were detected in almost all analyzed organs (Fig. 1d). Some genes were not detected in several organs by bulk RNA-seq, but were detected by reverse transcription PCR (RT-PCR) validation (as indicated by the asterisks in Fig. 1d and S2). Each chemoreceptor gene family has been subdivided into several subfamilies based on molecular phylogenetic analysis (Fig. 1e [33, 38, 63]). Conventional chemoreceptor genes have undergone repeated birth-and-death processes during evolution, forming species-specific gene repertoires. In contrast, the newly identified organ-independently expressed genes represent conserved subfamilies with stable gene numbers, mostly existing as single-copy genes across species, rather than the conventionally expanded genes. These results imply that while conventional-nasal/oral receptors have undergone evolutionary expansion to detect a wide range of chemical compounds, non-conventional extra-nasal/oral chemoreceptors have remained evolutionarily stable to detect specific chemical compounds. Among these, OR-κ was the only gene exhibiting multi-organ expression conserved across all four analyzed species, showing expression in all analyzed organs in three species (mouse, zebrafish and *Polypterus*) as well as expression in most of the organs analyzed in *Xenopus* (Fig. 1d, S2a and b). Based on this high degree of conservation, we focused on OR-κ as a representative example of a conservative chemoreceptor gene in the following analysis.

### Evolutionary characteristics of organ-independently expressed OR-κ genes

**Fig. 2:**
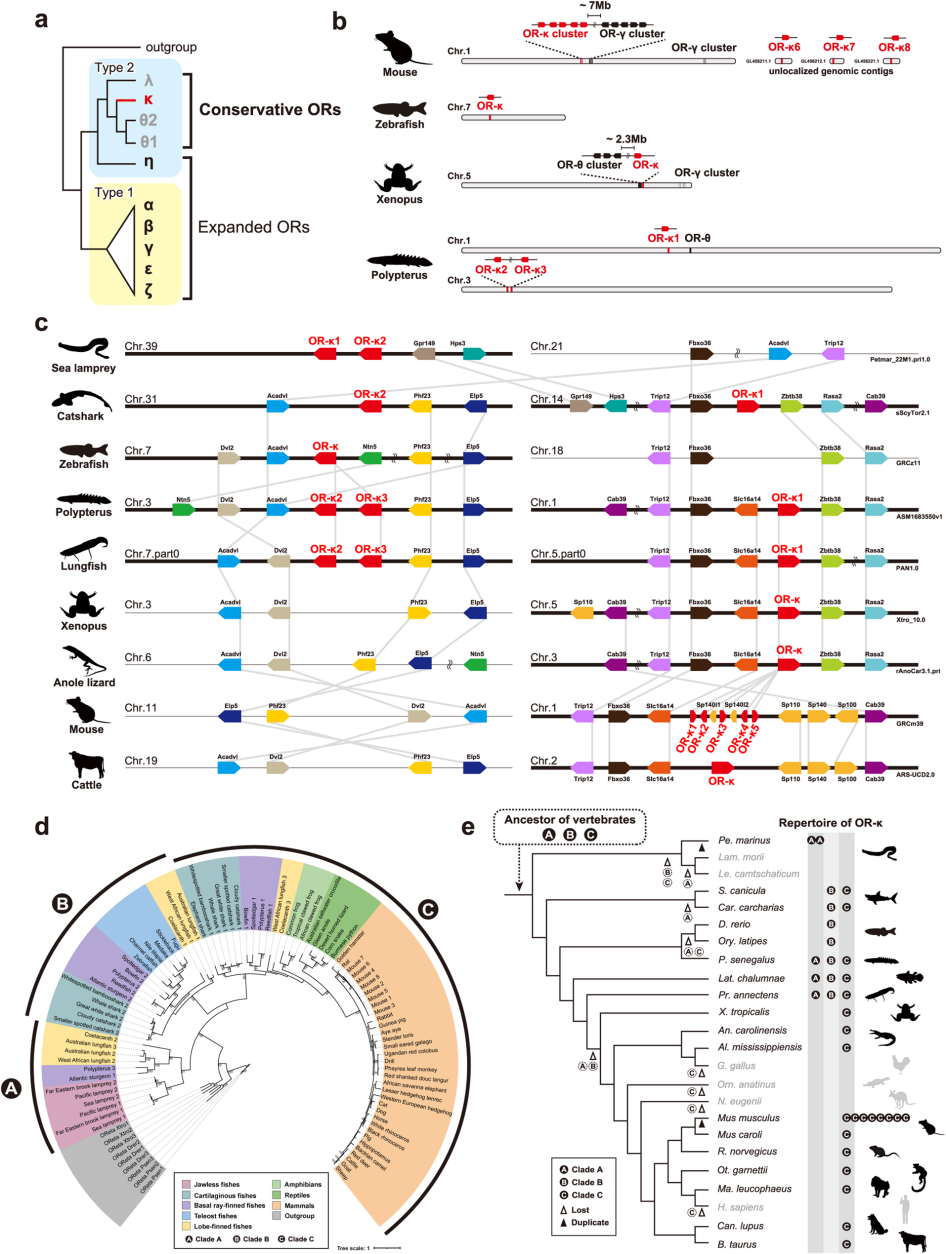
Genomic location and evolutionary history of OR-κ genes(a) Classification overview of each olfactory receptor (OR) gene subfamily. The classification scheme follows previous studies [121, 122]. The OR-κ investigated in this study belongs to the Type 2 group, representing an evolutionarily conserved OR subfamily. (b) Isolated genomic location of OR-κ genes. The genomic positions of OR-κ and other OR genes located on the same chromosome are shown for each species. Even in Xenopus, where OR-κ is closest to another OR (OR-θ), these two genes are separated more than 2.3 Mb, indicating that OR-κ is genomically isolated from other ORs. (c) Genomic synteny of OR-κ genes. The syntenic relationships surrounding OR-κ (red pentagon) are conserved within both the gill-breathing lineage (*e.g.*, catshark, zebrafish, *Polypterus*, and lungfish) and the air-breathing lineage (*e.g.*, *Polypterus*, lungfish, *Xenopus*, anole lizard, mouse, and cattle). Gene positions and orientations were obtained from NCBI RefSeq (https://www.ncbi.nlm.nih.gov/refseq/). (d) Maximum-likelihood tree for amino acid sequences inferred from 85 OR-κ for 58 vertebrates constructed with the JTT + G4 model in RAxML-NG. Three OR-η genes were randomly selected from each of Polypterus, zebrafish, and Xenopus and used as outgroups. Species classification is represented with colored highlighting at the tips of the tree. Bootstrap values from 100 resamplings are shown for clades with >80% support. OR-κ genes cluster into three major clades designated A, B, and C. (e) Schematic diagram of OR-κ gene duplication and loss during vertebrate evolution. The diagram illustrates OR-κ clades A, B, and C (black circles), gene duplication events (black arrowheads), and loss events (white arrowheads and dashed circles) across the vertebrate phylogeny. Some genus names are abbreviated; full details are provided in Table S6.

Next, we characterized the evolutionary features of OR-κ as a representative of organ-independently expressed chemoreceptor genes (Fig. 2). Olfactory receptors are known to be divided into two major clades through molecular phylogenetic analysis, Type 1 and Type 2 [38]. Type 1 ORs are composed of the expanded subfamily, exhibiting lineage-specific birth-and-death processes that enable the detection of diverse chemical substances. In contrast, Type 2 ORs, which include OR-κ, are composed of the conserved subfamilies with stable gene copy numbers, except for OR-η (Fig. 2a [38]). It is known that conventional expanded ORs are organized genomically to be co-regulated by shared transcriptional regulatory sequences such as the H element [64], the P element [65], the J element [66] and Greek islands [67]. Since the OR-κ gene was more than 2.3 Mb apart from other OR genes in all analyzed species, it is likely to be transcribed independently of the regulatory mechanisms controlling expanded OR genes (Fig. 2b). A similar genomic arrangement was also observed for other conservative ORs, such as OR-θ (Fig. 2b). Analysis of genomic synteny revealed that the OR-κ genomic region is conserved within both the gill-breathing lineage (*e.g.*, catshark, zebrafish, *Polypterus*, and lungfish) and the air-breathing lineage ( *e.g., Polypterus*, lungfish, *Xenopus*, anole lizard, mouse, and cattle), indicating an ancient evolutionary origin (Fig. 2c). Moreover, to clarify the phylogenetic relationships of OR-κ genes conserved within the two lineages identified by the synteny analysis (the gill-breathing and air-breathing lineage), we performed a molecular phylogenetic analysis. Phylogenetic reconstruction of OR-κ genes from 58 vertebrate species revealed the presence of three distinct clades, designated Clades A, B, and C (Fig. 2d). Clade A was conserved in jawless fishes, basal ray-finned fishes, and lobe-finned fishes; clade B in cartilaginous fishes, basal ray-finned fishes, teleost fishes, and lobe-finned fishes; and clade C in cartilaginous fishes, basal ray-finned fishes, lobe-finned fishes, amphibians, reptiles, and mammals. These results indicate that the common ancestor of vertebrates possessed at least three OR-κ genes, which subsequently experienced lineage- or species-specific gene losses and duplications during vertebrate evolution (Fig. 2e).

### Expression of OR-κ genes at single-cell resolution

**Fig. 3:**
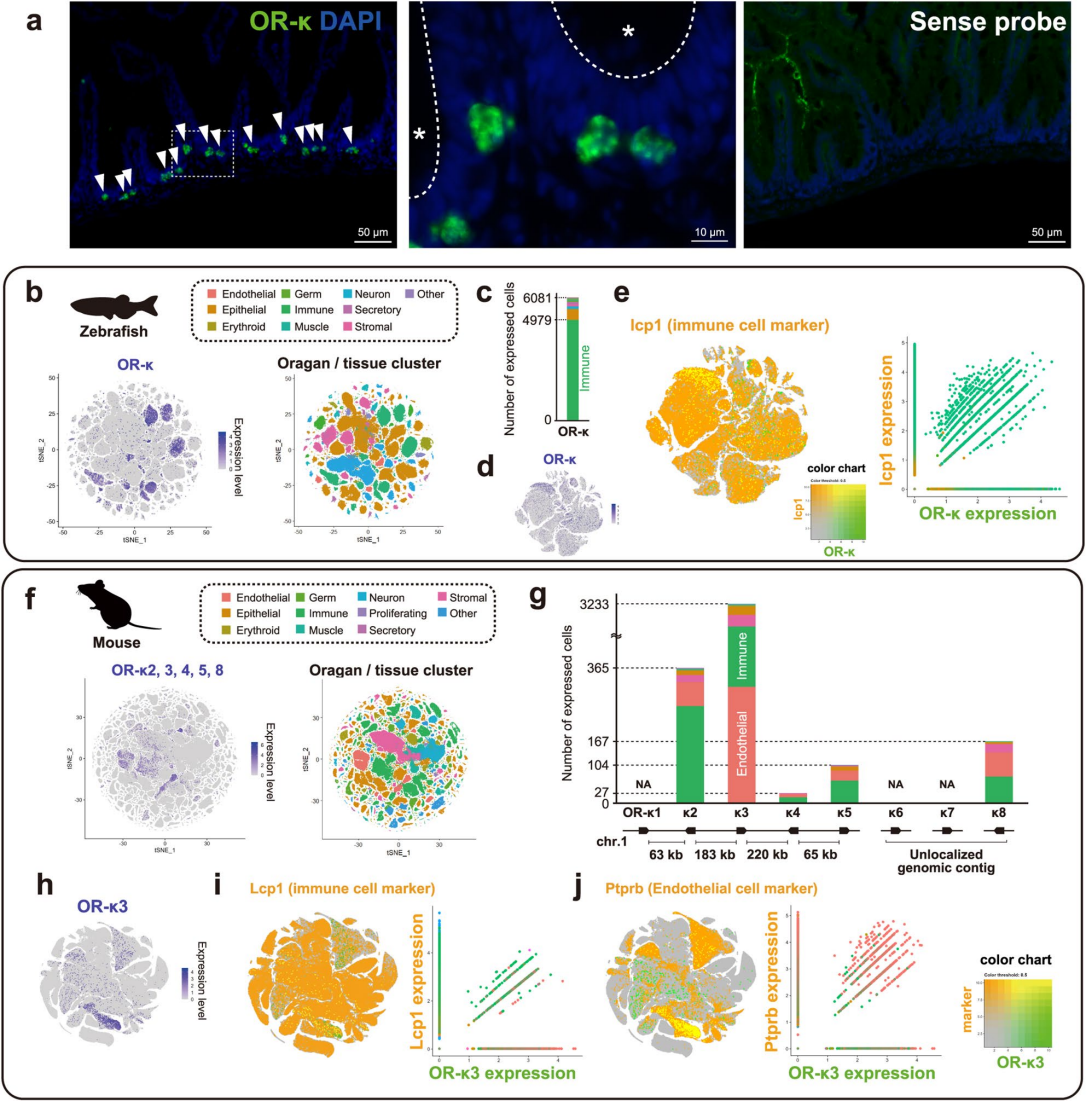
Expression of OR-κ genes in zebrafish and mouse at single cell resolution. (a)* in situ *hybridization analysis of OR-κ expression in the zebrafish intestine. Left panel shows the OR-κ expression pattern in the intestine. The detected signals were concentrated in the intestinal crypts (arrowheads). Middle panel shows a higher-magnification view of the white dotted box area in the left panel. The asterisk marks the intestinal lumen, and the dotted line marks the tissue boundary. Right panel shows the result of negative control using sense probe. (b–e) OR-κ gene expression revealed by re-analysis of single-cell RNA-seq data in zebrafish. (b) OR-κ expression pattern based on whole-body single-cell RNA-seq data. (c) Cell types of detected OR-κ-expressing cells and their proportions; bar colors follow Fig. 3b. OR-κ expression was predominantly detected in immune cells. (d–e) Re-analysis focusing exclusively on cell types extracted from the dataset used in Fig. 3c. (d) Expression levels of OR-κ genes in immune cells. (e) Co-expression of OR-κ and marker genes in immune cells; the marker gene expression shown in orange, OR-κ expression in green and co-expression in yellow. (f–j) OR-κ gene expression revealed by re-analysis of single-cell RNA-seq data in mouse. (f) OR-κ expression pattern based on single-cell RNA-seq data from 29 tissues. Results for genome-annotated OR-κ2, 3, 4, 5 and 8 are collectively displayed. (g) Cell types of detected OR-κ-expressing cells and their proportions; bar colors follow Fig. 3f. OR-κ expression was mainly detected in immune and endothelial cells. (h–j) Re-analysis focusing exclusively on immune and endothelial cells extracted from the dataset used in Fig. 3g. (h) Expression levels of OR-κ genes in immune and endothelial cells (representative result shown). (i–j) Co-expression of marker genes and OR-κ3 in immune (i) and endothelial (j) cells. Marker gene expression shown in orange, OR-κ3 expression in green and co-expression in yellow.

**Fig. 4:**
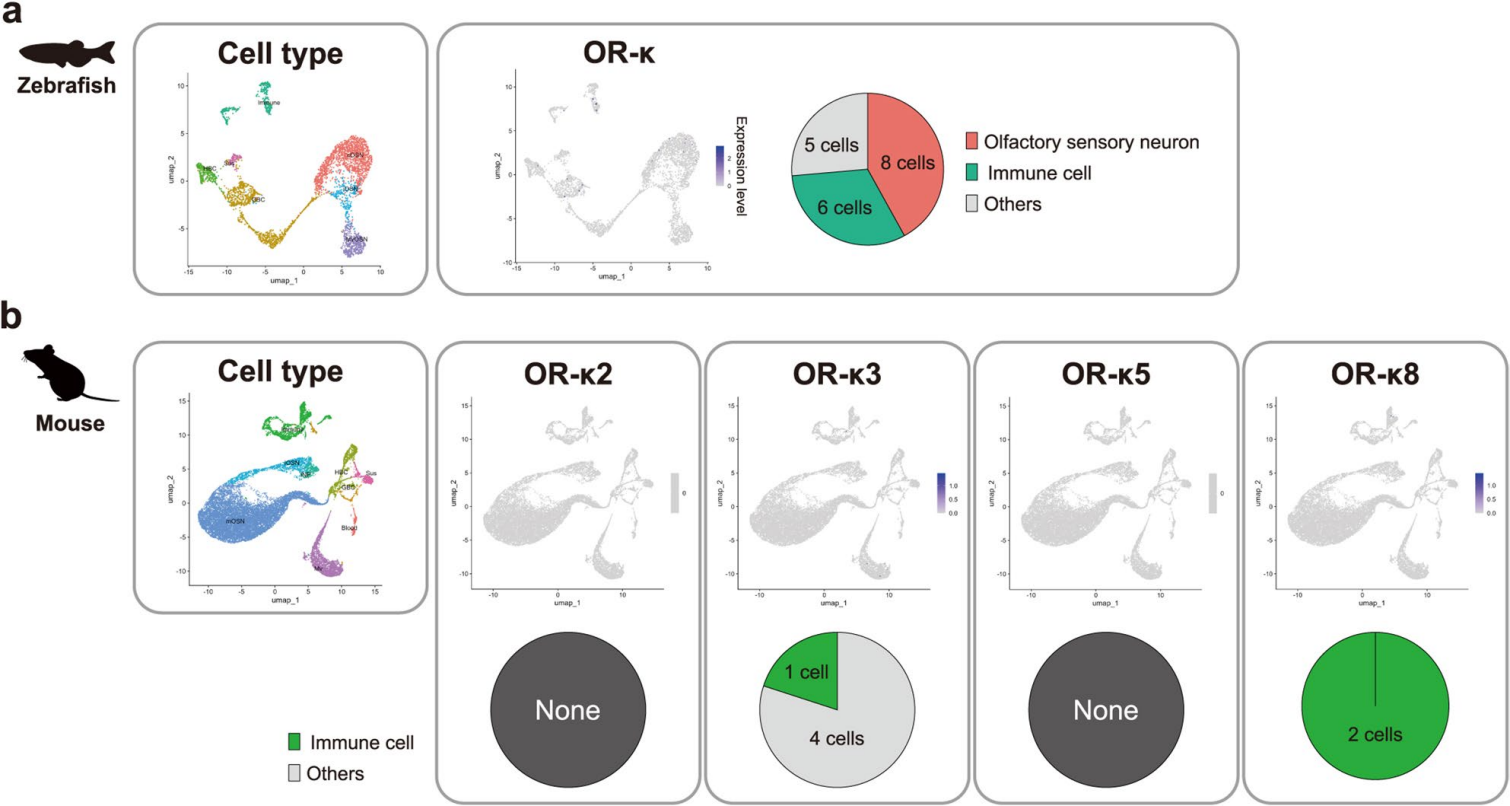
Expression of OR-κ genes in the olfactory organ. (a) Reanalysis of single-cell RNA-seq data from the zebrafish olfactory organ. OR-κ expression was detected in both olfactory sensory neurons and immune cells. (b) Re-analysis of single-cell RNA seq data from the mouse main olfactor y organ. No detectable expression of any OR-κ gene was observed in olfactory sensory neurons

Next, we identified the cell types expressing OR-κ genes (Fig. 3). We performed *in situ* hybridization and successfully visualized OR-κ-expressing cells in the intestine (Fig. 3a). The mRNA signal was concentrated in the basal region of intestinal folds and localized beneath the monolayered intestinal epithelium. To further characterize the OR-κ-expressing cells, we re-analyzed published single-cell RNA-seq datasets from zebrafish (Fig. 3b–e) and mouse (Fig. 3f–j). A comprehensive single-cell RNA-seq dataset of zebrafish encompassing whole-body tissues across developmental stages (pharyngula to adult) revealed OR-κ expression in multiple cell clusters (Fig. 3b), predominantly in immune cells (82%; 4979/6,081 cells, Fig. 3cand Table S7). Focused analysis of the immune cell clusters confirmed that OR-κ is co-expressed with canonical immune cell markers, such as *lcp1*, *ctss1*, *csf1ra*, *mpx* and *marco* [68–72] (Fig. 3d, e and S4a). These findings suggest that the organ-independent expression of zebrafish OR-κ is likely derived from immune cells. Re-analysis of single-cell RNA-seq data from 29 organs in mouse similarly revealed OR-κ expression across multiple cell clusters (Fig. 3f), particularly in immune cells (36.6%; 1,336/3,653 cells) and endothelial cells (51%; 1,869/3,653 cells) (Fig. 3g and Table S8). Consistent with bulk RNA-seq results, OR-κ expression was detected across cells from multiple tissues (Table S9). Analysis restricted to immune and endothelial cells demonstrated co-expression of OR-κ with known cell-type-specific markers, such as *Ptprb*, *Clec4g*, *Pecam1*, *Tie1* and *Cdh5* [73–75] (Fig. 3h–j and S4b). In contrast, OR-κ expression in immune cells was also detected in single-cell RNA-seq analysis of olfactory organs (Fig. 4). In the zebrafish olfactory epithelium, OR-κ expression was detected in 19 of 4,075 cells, including 8 olfactory sensory neurons and 6 immune cells (Fig. 4a and S3). In the mouse main olfactory epithelium (21,809 cells), OR-κ2 and OR-κ5 were not detected, whereas OR-κ3 and OR-κ8 were detected in only a few cells (5 and 2 cells, respectively). Notably, no OR-κ expression was observed in olfactory sensory neurons; instead, OR-κ3 and OR-κ8 signals were confined to immune cells (1 and 2 cells, respectively) (Fig. 4b).

### Broad tissue expression represents the ancestral state of vertebrate olfactory receptors

OR-κ genes are also present in the genomes of jawless fishes, such as lampreys and hagfish, which represent the most basal vertebrate lineage (Fig. 1e). To investigate the ancestral expression patterns of olfactory receptor (OR) genes in vertebrates, we examined OR gene expression in nasal and extra-nasal organs of lamprey using bulk RNA-seq analysis (Fig. S5a, b and Table S12). The total expression levels of all lamprey OR genes were highest in the nasal organ. However, OR-κ1 was expressed not only in the nasal organ but also in the brain and kidney, whereas OR-κ2 showed predominant expression in the liver, kidney, and ovary. Consistent with these patterns, many other OR genes (26/33 genes) were also detected in extra-nasal organs, suggesting that extra-nasal expression of OR genes represent a common feature in jawless vertebrates.

The cephalochordate amphioxus has occupies a key phylogenetic position for understanding the molecular evolution of vertebrates. Notably, amphioxus possesses a unique repertoire of OR genes that are distinct from both Type I and Type II vertebrate ORs, making it an informative model for examining the evolutionary dynamics of OR gene expression (Fig. S5c). Previous studies have reported that at least one amphioxus OR gene is expressed in bipolar neurons embedded within the rostral epithelium (rostrum), a putative sensory structure in adult amphioxus [76]. To comprehensively assess OR gene expression in amphioxus, we analyzed bulk RNA-seq data from 16 organs, including the rostrum, for all 38 identified amphioxus OR genes (Fig. S5d and Table S13). Five OR genes were detected in the rostrum; however, all of these genes were also expressed in other organs. Moreover, expression of the majority of amphioxus OR genes (79%; 30 of 38 genes) was detected exclusively in non-sensory tissues. These results suggest that most amphioxus OR genes primarily function in extra-sensory tissues rather than being restricted to sensory organs.

## Discussion

### Evolutionary dynamics of conventional (expanded) and conservative (stable) chemoreceptor genes

Vertebrate chemoreceptor genes constitute a multigene family that has undergone repeated gene duplication and pseudogenization (birth-and-death process) during evolution, resulting in lineage-specific diversity in gene copy number and sequence variation [37, 42]. Such gene turnover is thought to contribute to the accurate discrimination of diverse chemical cues from the external environment [42]. Therefore, gene number expansion through frequent tandem duplication within genomic clusters is interpreted as a typical evolutionary pattern of chemoreceptors [25, 77–79]. In contrast to these conventionally expanded genes, our analysis identified several evolutionarily stable chemoreceptor genes that exhibit organ-independent expression (Fig. 1d–e). Among them, the OR-κ gene was notably expressed in immune cells, and this expression pattern was shared between zebrafish and mouse (Fig. 3b–j), suggesting a conserved role of OR-κ gene vertebrate immune systems. Previous studies have reported that certain evolutionarily conserved ORs —such as *OR51E1*/*Olfr558* [52, 80] and *OR51E2*/*Olfr78* [53, 81]—function in extra-nasal organs. In addition, ORs conserved between human and chimpanzee also tend to be expressed extra-nasally [82]. Although the specific cell types expressing other evolutionary stable genes (e.g., OR-θ1, *ancV1R*, and *T1R1*) were not identified in this study, our findings suggest these receptors may serve functions distinct from odorant/gustatory perception. Further identification of their expressing cells will provide valuable insights into the molecular evolution and diversification of chemoreceptor gene functions.

### Evolution of OR gene expression patterns: from invertebrate chordates to basal vertebrates

We further explored the evolutionary origin of olfactory receptor (OR) gene expression patterns in vertebrates. Extra-nasal expression of OR genes has previously been reported in the hagfish (*Eptatretus burgeri*, inshore hagfish), a jawless vertebrate species. However, because hagfish have lineage-specifically lost Type 2 OR genes, they provide limited insight into the ancestral state of vertebrate OR repertoires [30]. To address this limitation, we focused on lampreys, which retain both Type 1 and Type 2 OR genes. In lampreys, expression of both Type 1 and Type 2 OR genes were detected in extra-nasal organs, and notably, several OR genes showed no detectable expression in the nasal organ (Fig. S5b). These results indicate that OR gene expression in jawless vertebrates is not restricted to the nasal organ, suggesting that organ-independent expression represents an ancestral feature rather than a derived condition.

To further trace the evolutionary origin of this expression pattern, we analyzed OR gene expression in an invertebrate chordate, the amphioxus (*Branchiostoma floridae*, florida lancelet), which occupies a key phylogenetic position for understanding vertebrate molecular evolution. Previous studies have reported OR gene expression in bipolar neurons embedded within the rostral epithelium (rostrum), a structure suggested to function as a chemosensory organ in adult amphioxus [76]. In our analysis, expression of five OR genes, including OR2 as a representative example, was detected in the rostrum. However, the majority of amphioxus OR genes were predominantly expressed in non-sensory tissues (Fig. S5d).

Together, these findings suggest that ancestral OR genes in early chordates and basal vertebrates were not exclusively specialized for chemosensory organs. Instead, OR genes likely exhibited broad, organ-independent expression patterns, with functional specialization toward sensory organs emerging later during vertebrate evolution. This evolutionary trajectory supports the view that olfactory receptors originally served more generalized physiological roles before being co-opted and refined for chemosensory perception in vertebrate nasal organs.

### Distinct transcriptional regulation of the OR-κ gene

The transcriptional regulatory mechanisms of conventional- (expanded-) chemoreceptor genes have been well characterized. These genes are often organized in genomic clusters, are controlled by shared enhancers [64–66, 83, 84], heterochromatin-mediated silencing [85, 86] and dynamic three-dimensional chromosomal rearrangements [67, 87, 88]. It has been proposed that extra-nasal expression of ORs may result from the leakage or relaxation of these regulatory constraints within expanded gene clusters [51]. Many such ORs maintain their original olfactory function while also acquiring novel physiological roles in other tissues [43, 51, 89–93]. Interestingly, the OR-κ gene identified in this study has been evolutionarily isolated from other OR gene clusters since its origin, remaining more than 2.3 Mb away from any other OR loci in all analyzed species (Fig. 2b and S4). Public genomic data from jawless fish further support this isolation, showing no other OR genes on the chromosome containing the OR-κ locus [37]. This consistent genomic isolation implies that OR-κ was transcribed under an independent regulatory mechanism distinct from that of conventional OR clusters at its origin. Such divergence in transcriptional control may underlie its unique expression in immune cells rather than olfactory sensory neurons. Notably, similar genomic isolation was also observed for other conservative ORs (e.g., OR-θ), raising the possibility that these evolutionarily stable genes may likewise be expressed extra-nasally under distinct transcriptional control. Future studies focusing on the transcriptional regulation of OR-κ and other evolutionarily stable chemoreceptor genes will be crucial for elucidating the evolutionary origins of regulatory mechanisms governing chemoreceptor gene expression.

### Possible functions of the OR-κ gene in immune and endothelial cells

Expression of OR genes in immune cells has been reported for several Type 1 ORs (conventional expanded ORs) in mouse [94–96], human, cattle [97], opossum and platypus [98]. In mice, these ORs have been implicated in cellular chemotaxis [94–96]. Such expression is thought to have arisen secondarily as a consequence of escaping the canonical transcriptional constraints during the birth-and-death evolution of expanded ORs. In contrast, comparative genomic studies of 2,210 vertebrate genome assemblies have demonstrated that OR-κ displays no evidence of large-scale gene duplication from jawless fishes to mammals [37]. Our bulk RNA-seq analyses detected OR-κ expression in multiple tissues, likely reflecting its presence in circulating leukocytes distributed throughout the body. Among leukocyte populations, T cells, macrophages, and eosinophils are known to be abundantly distributed in the lamina propria, a tissue layer directly beneath the intestinal epithelium [99, 100]. Based on this distribution pattern, the OR-κ-expressing cells detected by in situ hybridization (ISH) in the zebrafish intestine are presumably one of these immune cell types. Furthermore, the cell sizes of T cells (8–10 μm), macrophages (15–20 μm), and eosinophils (12–17 μm) are well characterized. The OR-κ-expressing cells visualized by ISH, which exhibited dimensions of approximately 8–13 μm, are consistent with these immune cell sizes, thereby suggesting that OR-κ is indeed expressed in these immune cell populations.

Although the precise immune function of OR-κ remains unclear, several results in this study provide intriguing clues. First, the evolutionary origin of OR-κ can be traced back to the common ancestor of vertebrates, including jawless fish (Fig. 1e), coinciding with the emergence of adaptive immunity [101, 102]. Second, in mice, OR-κ was also expressed in a substantial population of endothelial cells in addition to immune cells (Fig. 3f–j). Given that, immune and endothelial cells are thought to share both evolutionary [103–105] and developmental origins [106–110], the shared expression of OR-κ in these two cell types is consistent with this finding and offers valuable insight into its potential role. Finally, although the OR-κ gene is shared across most vertebrates, it has been lost independently in several lineages, including human, opossum, platypus, and chicken [34, 38]. Whether this loss of OR-κ affects immune function or is compensated by other OR genes remains an important question. Determining the precise evolutionary timing of OR-κ gene loss will provide further insight into its ancestral role. Because our analyses were limited to representative species, expanding taxonomic coverage will be essential for future analyses.

Taken together, the OR-κ gene appears to function differently from conventional olfactory receptor genes expressed in olfactory sensory neurons. Rather than contributing to the perception of external stimuli, OR-κ may play a role in sensing and regulating internal physiological or immune states. Comprehensive investigation of the evolutionary history, expression patterns, and molecular functions of OR-κ and other evolutionary stable chemoreceptors will deepen our understanding of the origin and diversification of chemoreceptor systems.

## Conclusion

In this study, we conducted a comprehensive survey of chemoreceptor gene expression across various extra-nasal and extra-oral organs in diverse vertebrate species, and evaluated their evolutionary dynamics. Notably, we identified the OR-κ, an olfactory receptor with stable gene numbers across vertebrates, as predominantly expressed in immune cell populations in both teleost fishes and mammals, unlike the conventional expanded OR families. Given that the OR-κ likely originated around the same evolutionary period as adaptive immunity—both emerging in the jawless vertebrate lineage—our results provide the first proposal for an evolutionarily conserved chemoreceptor expression in non-chemosensory organs across vertebrates. This work invites a reevaluation of chemoreceptor function, emphasizing their role beyond the classical olfactory and gustatory systems and implying an ancient link between chemosensation and the immune system conserved in vertebrates.

## Materials and methods

### Animals

*Polypterus* (*Polypterus senegalus*) were obtained from a commercial supplier (Nettaigyo-tsuhan Forest, Wakayama, Japan). The *Xenopus* (*Xenopus tropicalis*) strain Nigerian H (Xtr.NigerianH^Huarc^, RRID: HUARC_1002) and the zebrafish (*Danio rerio*) strain RIKEN WT (RW) were provided by National BioResource Project (NBRP) of MEXT through Hiroshima University Amphibian Research Center (RRID: SCR_019015) and RIKEN Center for Brain Science, respectively. All animals were maintained and bred at 27 °C on a 12/12 h light/dark cycle. All experiments were conducted in accordance with the Institutional Animal Experiment Committee of the Institute of Science Tokyo.

### Bulk RNA-seq analysis

RNA was extracted from *Polypterus* (intestine and pectoral fin) and *Xenopus* (nasal tissue, oral tissue, spinal cord, lung, heart, intestine, liver and kidney) using TRI Reagent (Molecular Research Center, Cincinnati, OH, USA). The extracted total RNA was sequenced at 100 bp paired-end reads on a NovaSeq 6000 or Novaseq X by Macrogen Japan Corp., using a TruSeq stranded mRNA Library Prep Kit. *Polypterus* kidney sequence data were unpublished, provided by Y. Kimura. All other RNA sequence data were obtained from the NCBI SRA database (Table S1). SRA files were retrieved and converted to FASTQ format using the SRA Toolkit prefetch and fastq-dump v3.0.7 [111]. The quality control of raw sequence data was performed with fastp v0.23.4 [112] with the following options: -q 20 -l 25. The reads were mapped to the genome of *Polypterus* (ASM1683550v1), zebrafish (GRCz11), *Xenopus* (UCB_Xtro_10.0), mouse (*Mus musculus*, GRCm39), lamprey (*Petromyzon marinus*, UKy_Petmar_22M1.pri1.0), or amphioxus (*Branchiostoma floridae*, Bfl_VNyyK) using STAR version 2.7.5c [113] and quantified using rsem-calculate-expression v1.3.3 [114]. The gene annotation data for the quantification were downloaded from NCBI Reference Sequence Database (RefSeq) and edited for the regions of known chemoreceptor genes [37]. We newly identified intact OR genes in lamprey and amphioxus by performing homology searches with amino acid sequences as queries, utilizing our original pipeline software FATE (https://github.com/Hikoyu/FATE), due to the use of different genome assembly versions in prior studies [37, 38]. NCBI database designations for the OR-κ genes are as follows: *zgc:194312* in zebrafish; *zgc:194312* (OR-κ2) and *olfactory receptor 4K13-like* (OR-κ3) in *Polypterus*; *Gm7582* (OR-κ2), *Gm7609* (OR-κ3), *Gm2666* (OR-κ4), *Gm7592* (OR-κ5), and *Csprs* (OR-κ8) in mouse. Following previous studies with human, the threshold for classifying a gene as expressed was defined at 0.01 TPM [49].

### Single-cell RNA-seq reanalysis

Public single-cell RNA-seq datasets reanalyzed in this study can be found on the Gene Expression Omnibus (GEO): GSE198832 [115]. Annotation of cell lineage and cell type for each cell followed Wang and colleagues [115]. Gene expression matrices from immune and endothelial cells in mice and immune cells in zebrafish were analyzed using Seurat v5 [116]. Twenty principal components (PCs) were selected as significant components for t-SNE analysis. The clustering parameter resolution was set to 0.8 for identifying cell clusters.

Raw single-cell RNA sequencing data from zebrafish olfactory epithelium were retrieved from the NCBI SRA database (SRR31595086 [117]). Fastq files were processed using 10x Genomics Cell Ranger v6.0.2 with the GRCz11_v4.3.2_cellranger_v6 transcriptome reference [118]. Quality control filtering was applied using the following thresholds: nCount_RNA > 250 and < 5000, and mitochondrial gene percentage < 15%. Data normalization and identification of highly variable features were performed using default parameters with the NormalizeData and FindVariableFeatures functions in Seurat. UMAP dimensionality reduction was performed using seven PCs with a resolution parameter of 0.8. Initial clustering identified 19 cell clusters, which were annotated into seven cell types based on marker gene expression patterns consistent with those reported by Chen and colleagues [117]. Single-cell RNA-seq data from mouse main olfactory epithelium were obtained from the GEO database (GSE185251 [119]). The filtered feature-barcode matrix was loaded into Seurat for downstream analysis. Data processing and cell type annotation were performed using identical parameters and marker genes as described in the original study.

### In situ hybridization (ISH)

ISH was performed according to the method of Suzuki et al. [120] with several modifications. Briefly, the entire intestines were fixed in Davidson’s solution overnight at 4 °C. The fixed tissues were then immersed in 20% sucrose in PBS overnight and embedded in optimal cutting temperature (O.C.T.) compound (Sakura Finetek Japan, Tokyo, Japan) before being frozen in liquid nitrogen. The embedded blocks were then sliced into Sects. 12 µm thick and placed on a coated glass slide. The frozen sections were then digested with 5 µg/ml proteinase K for 10 min at 37 °C, after which they were hybridized with 5 ng/µl DIG-labelled riboprobes at 65 °C overnight. The sections were washed, treated with 2 µg/ml RNase A in TNE buffer for 30 min at 37 °C. Then, they were treated with a streptavidin/biotin blocking kit (Vector Laboratories, Newark, CA, USA). After that, they were treated with 1% blocking reagent (PerkinElmer, Waltham, MA, USA) in TBS buffer for 1 h. Signals were detected with a peroxidase-conjugated anti-DIG antibody (1:100, 11,207,733,910, Roche), amplified by a TSA Plus Biotin kit (1:50, PerkinElmer), and visualized with an Alexa Fluor 488-conjugated streptavidin (1:200, Thermo Fisher Scientific, Waltham, MA, USA). The sections were mounted using Vectashield mounting medium containing DAPI (Vector Laboratories) and digitally captured using a Zeiss Axioplan SP fluorescence microscope and a Zeiss Axiocam 503 colour CCD camera (Carl Zeiss, Oberkochen, Germany).

### Reverse transcription-PCR (RT-PCR)

Total RNA was extracted from a panel of *Xenopus*, mouse, *Polypterus* and zebrafish target tissues. After DNase I digestion (TaKaRa, Shiga, Japan), RNA samples were diluted to 10 ng/μl, and cDNA was synthesized from 2 μg of total RNA using SuperScript III Reverse Transcriptase (Thermo Fisher Scientific) with oligo-dT18 primers. All PCRs were performed using KOD FX Neo polymerase or KOD One (TOYOBO, Osaka, Japan) with the following cycling conditions: 98 °C for 10 sec, 55 °C for 30 sec, and 68 °C for 30 sec, repeated for 35 cycles. Primer sequences are listed in Table S10. β-actin was used as an internal control for RNA quality and expression normalization in the chemosensory organ.

### Phylogenetic analysis

We obtained the full-length amino acid sequences of 85 OR-κ genes from 58 vertebrate species—comprising three jawless fishes, six cartilaginous fishes, five basal ray-finned fishes, six teleost fishes, one coelacanth, two lungfishes, three amphibians, five reptiles, and 27 mammals—from previous studies [37]. As an outgroup, we randomly selected three OR-η sequences each from *Polypterus*, zebrafish, and the *Xenopus*, which were retrieved using the same procedure. These sequences were aligned using MAFFT online v0.7 [121] with default parameters. Maximum likelihood (ML) phylogenetic tree was inferred using RAxML-NG v0.1.2.0 [122] with 100 bootstrap replicates, employing the optimal substitution model JTT+G4 as determined by ModelTest-NG v0.0.2.0 [123, 124]. ML phylogenetic tree was visualized with iTOL [125, 127].

## Electronic supplementary material

Below is the link to the electronic supplementary material.

Supplementary Tables 1–13

Supplementary Figures 1–5

## Data Availability

All sequence reads were deposited in the DDBJ Sequence Read Archive under accession no. PRJDB39629 and PRJDB39651.
