# Supplementary material for: Vertebrate-wide transcriptomic screening identifies immune cell-specific expression of the conserved OR-κ gene
Source: Zoological Lett. 2026 Feb 7;12:9. doi: 10.1186/s40851-026-00260-z (PMC13113109; doi:10.1186/s40851-026-00260-z)
Supplement: Supplementary file 2 — Supplementary Figures 1–5 [file 40851_2026_260_MOESM2_ESM.pdf]

This PDF file includes:

Supplementary Figures 1 –5

Other supplementary materials for this manuscript include the following:

Supplementary Tables 1–13

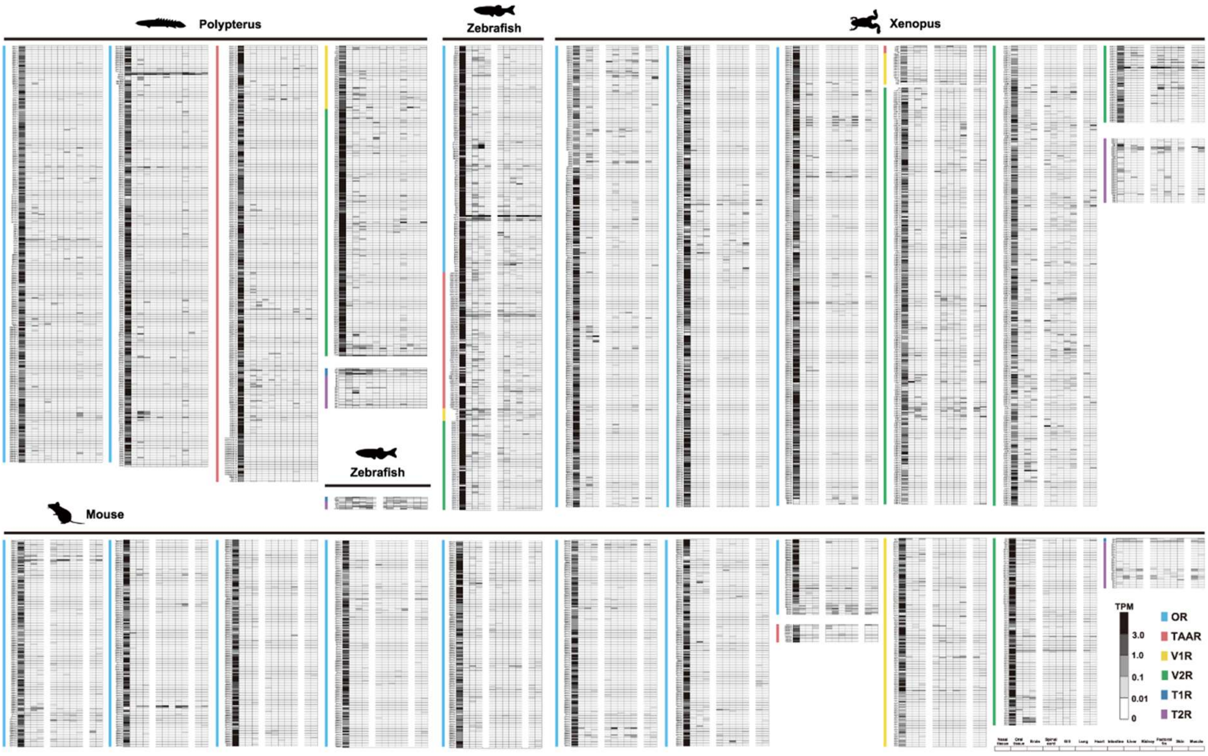

Fig. S1 | Heatmap showing gene expression profiles of all chemoreceptor genes analysed in this study.

Each row represents a chemoreceptor gene, and each column corresponds to an organ analysed. Normalized expression levels (TPM) are shown as a heatmap. The legend provided in the lower right corner indicates expression values, colour codes for chemoreceptor gene families, and the list of analysed organs.

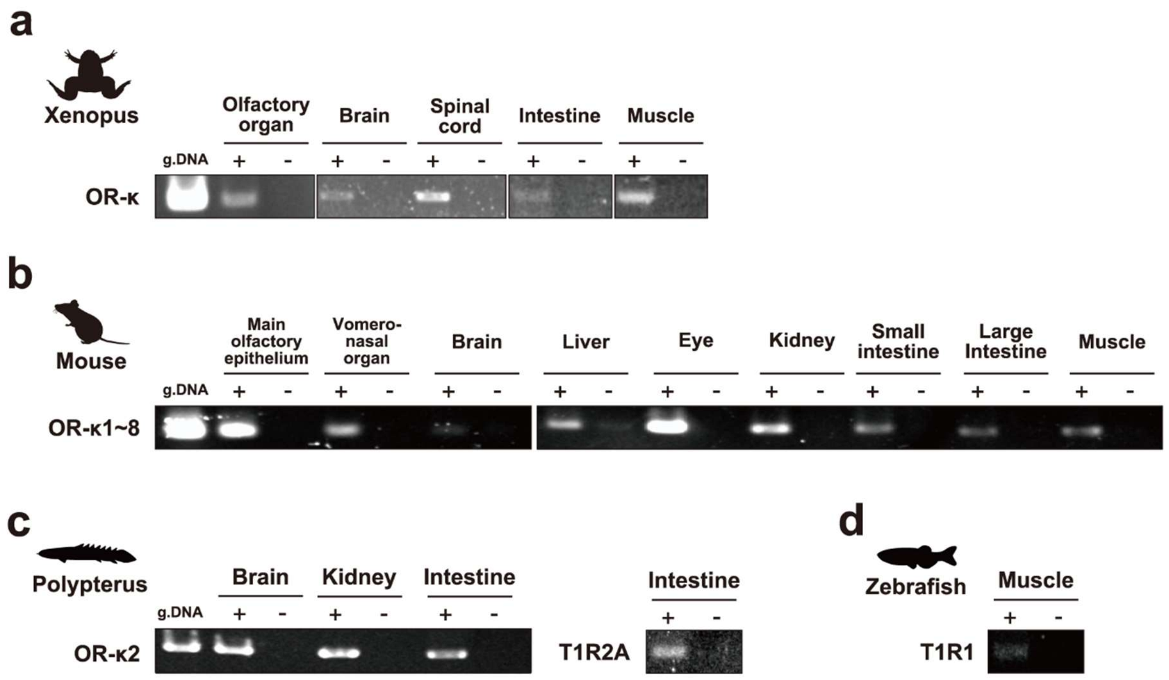

Fig. S2 | Reverse transcription PCR (RT-PCR) validation of chemoreceptor gene expression.

RT-PCR analysis validating chemoreceptor gene expression in tissues where transcripts were undetected or insufficiently represented by bulk RNA-seq analysis. OR-κ expression in various tissues of (a) *Xenopus*, (b) mouse, and (c) *Polypterus*, with *T1R2A* also detected in *Polypterus* intestine. (d) *T1R1* expression in zebrafish muscle. Genomic DNA (g.DNA) was used as a positive control. Because OR-κ, *T1R2A*, and *T1R1* genes consist of a single exon, RT-PCR was performed with and without reverse transcriptase (+/–) to distinguish cDNA-derived amplification products.

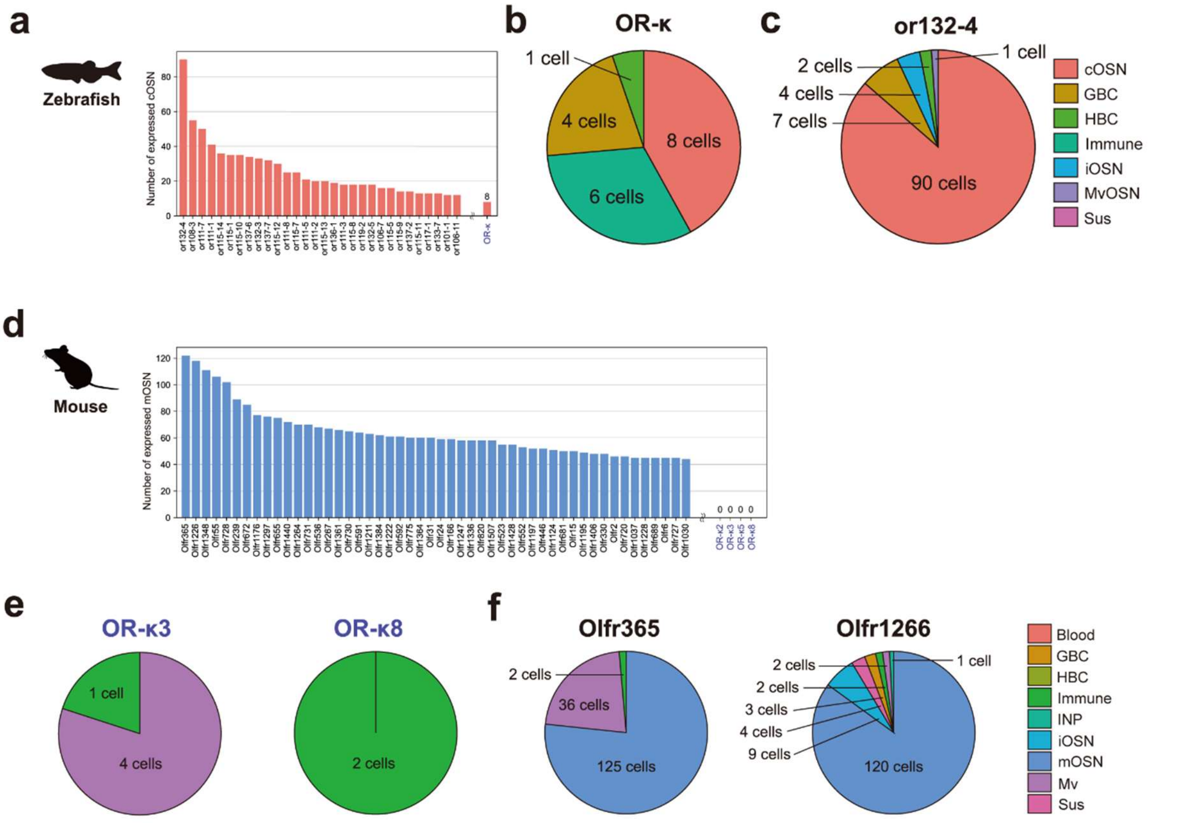

Fig. S3 | Expression profile of OR-κ genes revealed by single-cell RNA-seq analysis of the olfactory organ.

Expression patterns of OR-κ genes and representative canonical ORs in the zebrafish olfactory epithelium (a–c) and mouse main olfactory organ (d–f). (a) Number of cells expressing each OR gene in zebrafish ciliated olfactory sensory neurons (cOSNs). The top 30 canonical ORs with the highest number of expressing cells, together with OR-κ, are shown. As reported previously [21], canonical ORs are abundantly expressed in cOSNs. Consistent with this pattern, OR-κ was also detected in a small population of cOSNs (8 cells). (b, c) Cellular distribution of OR-κ–expressing cells (b) and a representative canonical OR (or132-4) (c). Similar to or132-4, OR-κ was detected in sOSNs (8 cells). In addition, OR-κ showed expression in immune cells, which was not observed for the canonical OR. cOSN: ciliated olfactory sensory neuron, GBC: globose basal cells, HBC: horizontal basal cells, iOSN: immature olfactory sensory neuron, MvOSN: microvillous olfactory sensory neuron, Sus: sustentacular cells. (d) Number of cells expressing each OR gene in mouse mature olfactory sensory neurons (mOSNs). The top 50 canonical ORs and all genome-annotated OR-κ genes are shown. As established in previous studies [126, 127], canonical ORs are broadly expressed in mOSNs, whereas none of the OR-κ genes were detected in mOSNs. (e, f) Cellular distribution of cells expressing OR-κ (OR-κ3 and OR-κ8) (e) and representative canonical ORs (Olfr365 and Olfr1266) (f). Canonical ORs showed robust expression in mOSNs (125 and 120 cells), whereas OR-κ expression was absent from this neuronal population. INP: immediate neuronal precursors, Mv: microvillar cells.

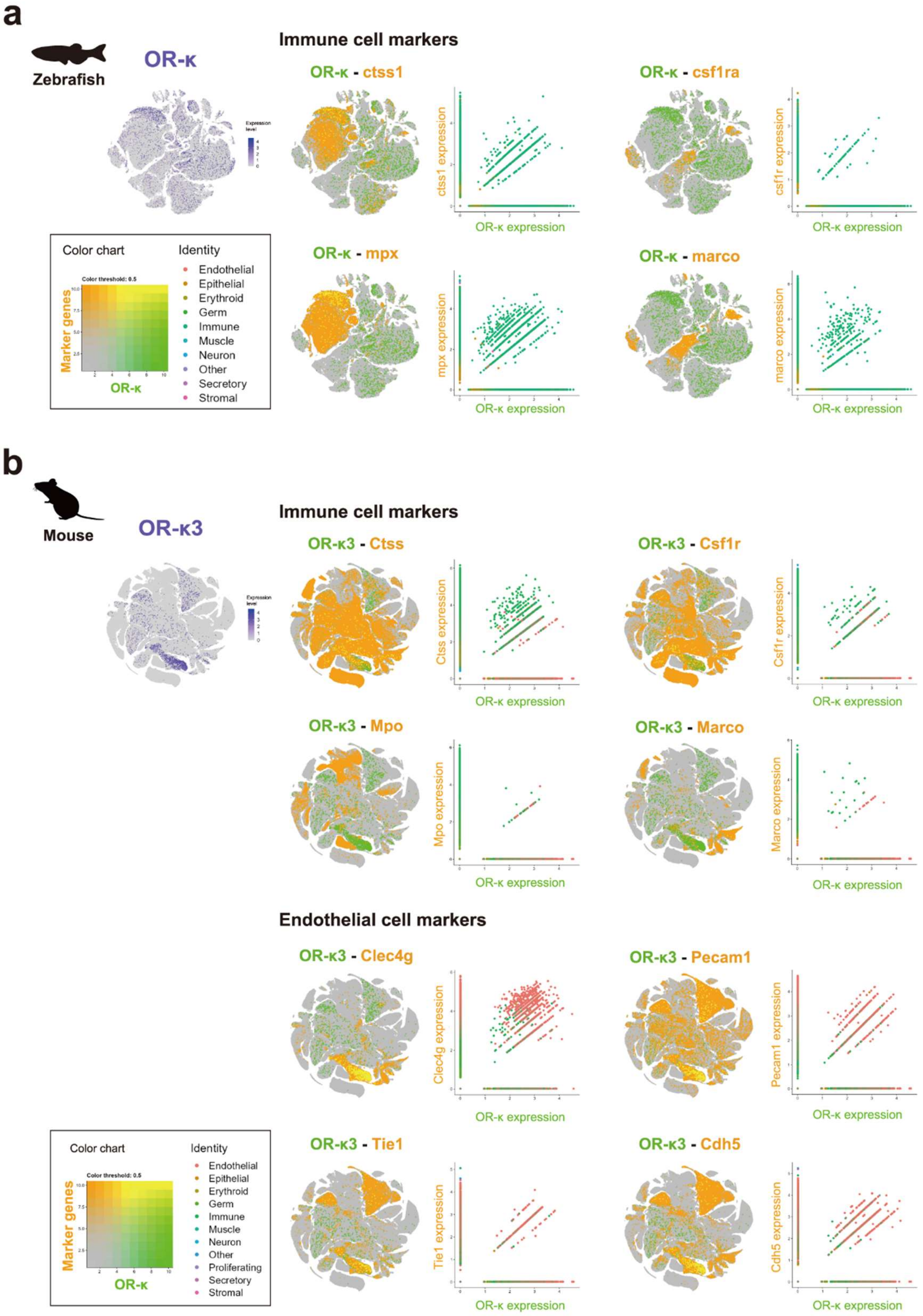
a
Zebrafish
OR-κ
Immune cell markers
OR-κ - ctss1
OR-κ - csf1ra
OR-κ - mpx
OR-κ - marco
ctss1 expression
csf1r expression
mpx expression
marco expression
OR-κ expression
Color chart
Color threshold: 0.5
Marker genes
OR-κ
Identity
Endothelial
Epithelial
Erythroid
Germ
Immune
Muscle
Neuron
Other
Secretory
Stromal
b
Mouse
OR-κ3
Immune cell markers
OR-κ3 - Ctss
OR-κ3 - Csf1r
OR-κ3 - Mpo
OR-κ3 - Marco
Ctss expression
Csf1r expression
Mpo expression
Marco expression
OR-κ expression
Endothelial cell markers
OR-κ3 - Clec4g
OR-κ3 - Pecam1
OR-κ3 - Tie1
OR-κ3 - Cdh5
Clec4g expression
Pecam1 expression
Tie1 expression
Cdh5 expression
Color chart
Color threshold: 0.5
Marker genes
OR-κ
Identity
Endothelial
Epithelial
Erythroid
Germ
Immune
Muscle
Neuron
Other
Proliferating
Secretory
Stromal

Fig. S4 | Co-expression of OR-κ and immune cell marker genes.

Single-cell RNA-seq datasets were re-analysed to visualize the co-expression of OR-κ and immune cell marker genes in (a) zebrafish and (b) mouse. Using the immune cell populations extracted from the datasets analysed in Fig. 3, OR-κ expression (green) and the expression of each immune cell marker gene (orange) were mapped onto t-SNE plots. Scatter plots show the co-expression relationships between OR-κ and the corresponding marker genes. Each dot represents a single cell, with OR-κ expression on the x-axis and marker gene expression on the y-axis. The linear distribution of points indicates strong co-expression of OR-κ with each marker gene. Cell-type identities are indicated by the colour scheme shown in the box on the left. In zebrafish, OR-κ expression was exclusively detected in immune cells, whereas in mouse, it was observed not only in immune cells but also in a substantial number of endothelial cells.

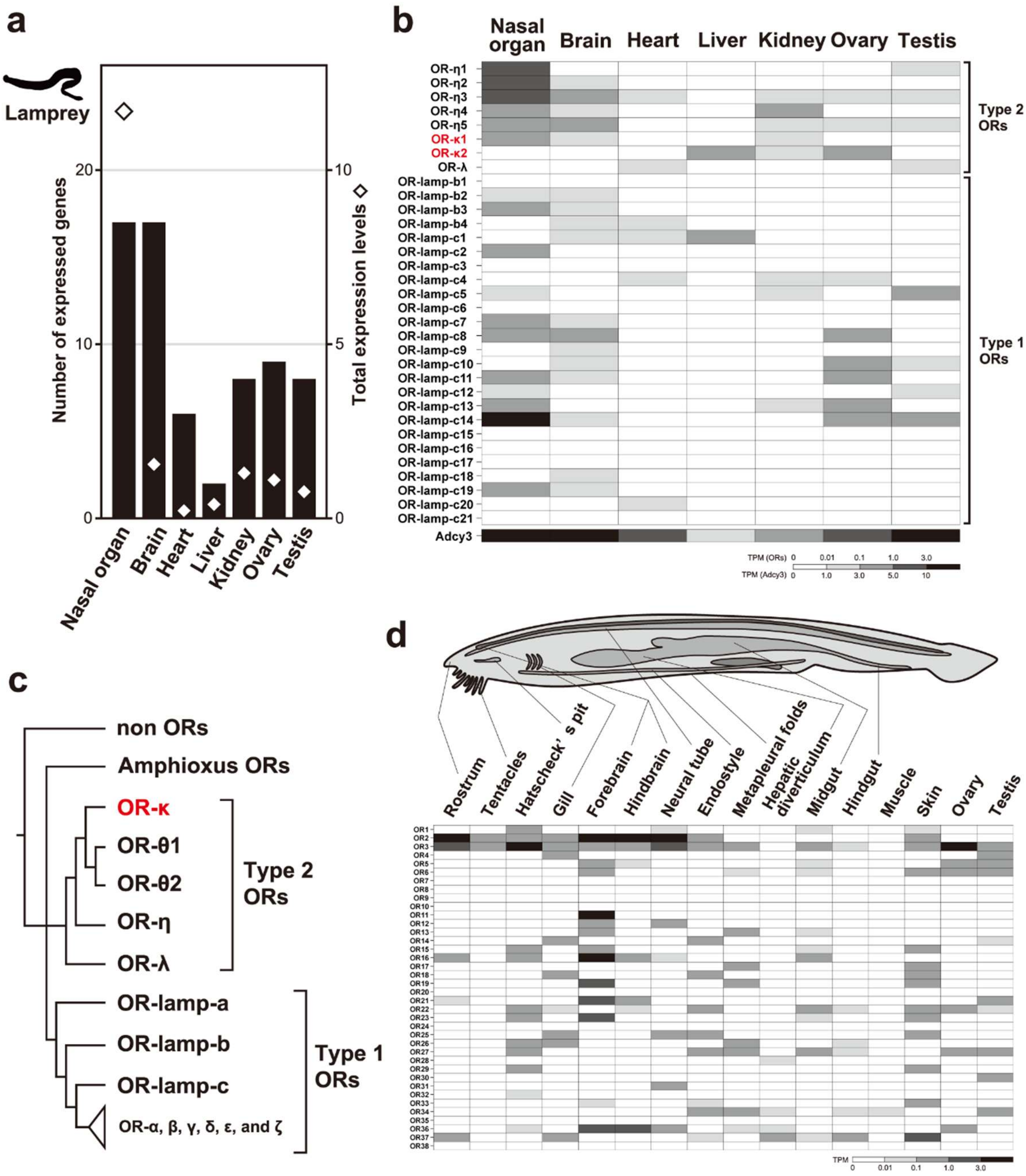

Fig. S5 | Expression of OR genes in various lamprey and amphioxus organs.

(a) Numbers and total expression levels of OR genes detected in nasal and extra-nasal organs. (b) Gene expression profiles of 33 lamprey OR genes in the nasal organ and six extra-nasal organs. As a positive control, the expression levels of the adenylate cyclase 3 gene (Adcy3), known to be distributed in the nasal organ, are shown below. (c) Phylogenetic relationships of OR genes based on previous studies [37, 38, 76]. The phylogenetic relationship between the amphioxus ORs and Type 1 and 2 ORs remains unclear. (d) Gene expression profiles of 38 amphioxus OR genes in various organs.
